# Sequence Design of Random Heteropolymers as Protein Mimics

**DOI:** 10.1021/acs.biomac.2c01036

**Published:** 2023-01-13

**Authors:** Ivan Jayapurna, Zhiyuan Ruan, Marco Eres, Prajna Jalagam, Spencer Jenkins, Ting Xu

**Affiliations:** † Department of Materials Science and Engineering, University of California, Berkeley, Berkeley, California 94720, United States; ‡ Department of Chemistry, University of California, Berkeley, Berkeley, California 94720, United States; § Materials Sciences Division, Lawrence Berkeley National Laboratory, Berkeley, California 94720, United States

## Abstract

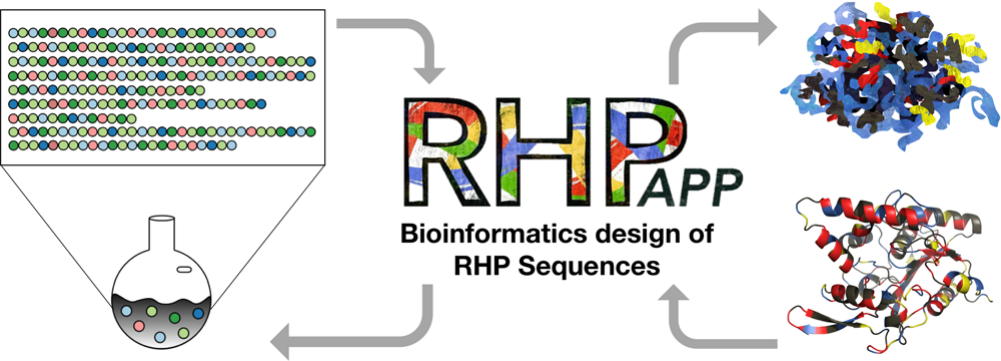

Random heteropolymers (RHPs) have been computationally designed and experimentally shown to recapitulate protein-like phase behavior and function. However, unlike proteins, RHP sequences are only statistically defined and cannot be sequenced. Recent developments in reversible-deactivation radical polymerization allowed simulated polymer sequences based on the well-established Mayo–Lewis equation to more accurately reflect ground-truth sequences that are experimentally synthesized. This led to opportunities to perform bioinformatics-inspired analysis on simulated sequences to guide the design, synthesis, and interpretation of RHPs. We compared batches on the order of 10000 simulated RHP sequences that vary by synthetically controllable and measurable RHP characteristics such as chemical heterogeneity and average degree of polymerization. Our analysis spans across 3 levels: segments along a single chain, sequences within a batch, and batch-averaged statistics. We discuss simulator fidelity and highlight the importance of robust segment definition. Examples are presented that demonstrate the use of simulated sequence analysis for in-silico iterative design to mimic protein hydrophobic/hydrophilic segment distributions in RHPs and compare RHP and protein sequence segments to explain experimental results of RHPs that mimic protein function. To facilitate the community use of this workflow, the simulator and analysis modules have been made available through an open source toolkit, the RHPapp.

## Introduction

Utilizing and mimicking protein function is a key approach to unlocking advanced, robust, cheap, and scalable functional materials. Heteropolymers are routinely used for surfactants,^1−5^ hydrogels,^6,7^ polyelectrolytes,^8−10^ gene delivery,^11−13^ and more.^14−17^ Chemistry diversification through monomer increments, side-chain modifications, or block copolymerization have been unsystematically explored as the primary design criteria for material functionalization. However, a more general chemical heterogeneity framework for rational design of protein-like heteropolymers is still lacking. Random heteropolymers (RHPs) are composed of more than two monomers, with sequences that are statistically defined. In comparison to proteins, RHPs are synthetic, and polydisperse in molecular weight and composition. RHPs can have batch-to-batch variations but cannot yet be sequenced with monomeric specificity. Despite key differences, several computational and experimental studies have demonstrated the ability for RHPs to recapitulate protein-like behaviors.^18−30^ Unlike sequence-specific heteropolymers,^31−34^ the lack of RHP sequence information significantly hampers our ability to further leverage the full potential of this unique class of polymers for precisely tailored functionality. Synthetic breakthroughs in reversible-deactivation radical polymerization (RDRP) have made it possible to synthesize heteropolymers with improved reproducibility and control over the probability of each monomer along the polymer chain.^35−43^ This narrows the gap between theoretically ideal polymerization and synthesized heteropolymers.

Since the 1940s there have been numerous efforts to simulate heteropolymers using experimental inputs such as monomer concentrations and reactivity ratios.^44−54^ Among those, Compositional Drift, a Monte Carlo method RDRP simulator, has been developed to simulate RHP sequences based on the Mayo–Lewis model.^55^ RDRP synthesis input parameters provide direct handles to tune key batch level characteristics such as monomer composition and average degree of polymerization. Synthetic control of batch level properties can be experimentally verified using common instrumentation such as nuclear magnetic resonance spectroscopy (NMR) and gel permeation chromatography (GPC). Matching these experimentally measurable, batch level, key characteristics in reality and theory allows for simulated outputs to be useful once abstracted from the level of monomeric precision to an analysis on the batch level of statistical distributions and sequence patterns. Prior works have shown the use of RDRP synthetic parameters to design RHPs with statistically controlled sequences as protein mimics with a wide variety of promising applications inclusive of enzyme stabilization, biodegradable plastics, and selective ion transport.^4,6,10,25,26,55,56^

Sequence analysis is routinely performed for proteins to evaluate statistical distributions of residues along a chain, identify key motifs, and assess similarity across proteins.^57−62^ Applying similar analysis to RHPs will advance our ability to design functional polymeric materials. Here we demonstrate a bioinformatics-inspired sequence analysis on batches of simulated RHP sequences. Specifically, we present the RHPapp, a more comprehensive version of the Compositional Drift simulation software, integrated into a suite of analytical modules for RHP design and analysis. Through the RHPapp, the synthesis of batches of RHPs with varying experimentally measurable, batch level characteristics are simulated. Common methods such as binarizing sequences by hydrophobicity and plotting hydropathy are applied to the simulated RHP sequences. To fully understand the heterogeneity of RHPs, we highlighted the importance of analyzing simulated outputs at multiple levels of abstraction: single chain segments (segment level), across sequences (sequence level), and across batches (batch level). Current results revealed that given appropriate evaluation metric selection and segment length definition, sequence analysis on simulated RHP sequences can help to rationalize experimental findings, guide subsequent experimental design in an iterative fashion, and realize designed function without the need for full sequence specificity or sequencing technology.

## Methods

We simulated RHP polymerization over ranges of values for each input parameter to RHPapp and compared batches across various modular metrics for evaluation and 3 levels of statistical heterogeneity: (1) single chain segments (segment level), (2) across sequences (sequence level), and (3) across batches (batch level). Target % conversion and polydispersity (PDI) of batches of simulated RHP sequences are tunable parameters. However, for all RHP sequences in this work conversion is fixed to 50% and polydispersity (PDI) is kept low (below 1.2) to reflect previous experimental results. Reactivity ratios used in this work are presented in Table S1. All oligomers (sequences of degree of polymerization (DP) < 15) are neglected, as they are removed experimentally in the purification process of RHP synthesis, as is described in prior works.^25^ Currently, 4 evaluation metrics have been implemented as Python modules, with which RHP sequences are analyzed. These metrics are modular; thus, the addition of new metrics as future work is straightforward.

**Figure 1:**
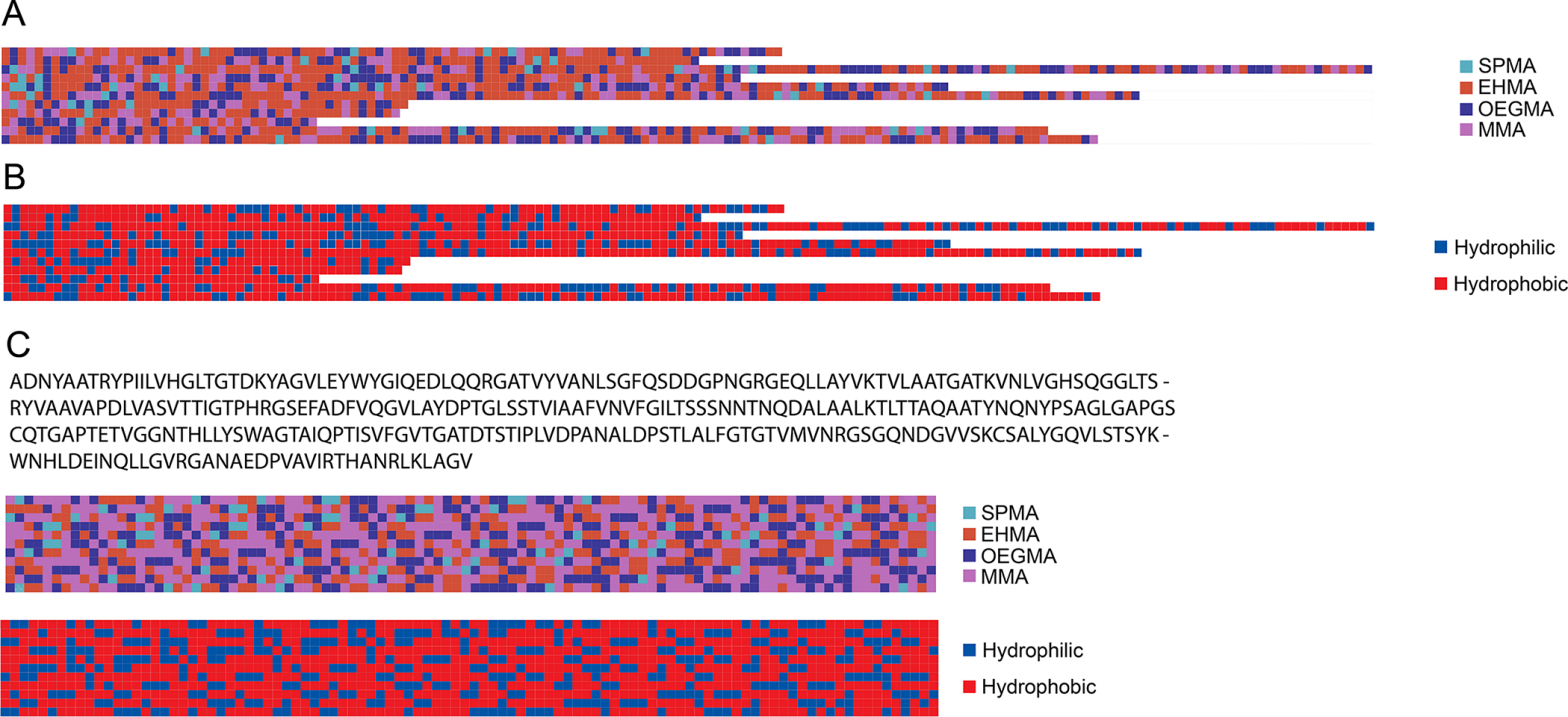
Random heteropolymer (RHP) and protein sequence comparison. (A) Random sequences sampled from a simulated 4 monomer RHP of 50% methyl methacrylate (MMA), 25% poly(ethylene glycol) average *M*_n_ 500 (OEGMA500), 20% 2-ethylhexyl methacrylate (EHMA), and 5% 3-sulfopropyl methacrylate potassium salt (SPMA) (B) RHP sequences binarized to hydrophobic and hydrophilic. (C) Full sequence for*Burkholderia cepacia*lipase (BC-Lip), segmented and translated to RHP sequence space and then binarized to hydrophobic and hydrophilic units.

**Figure 2:**
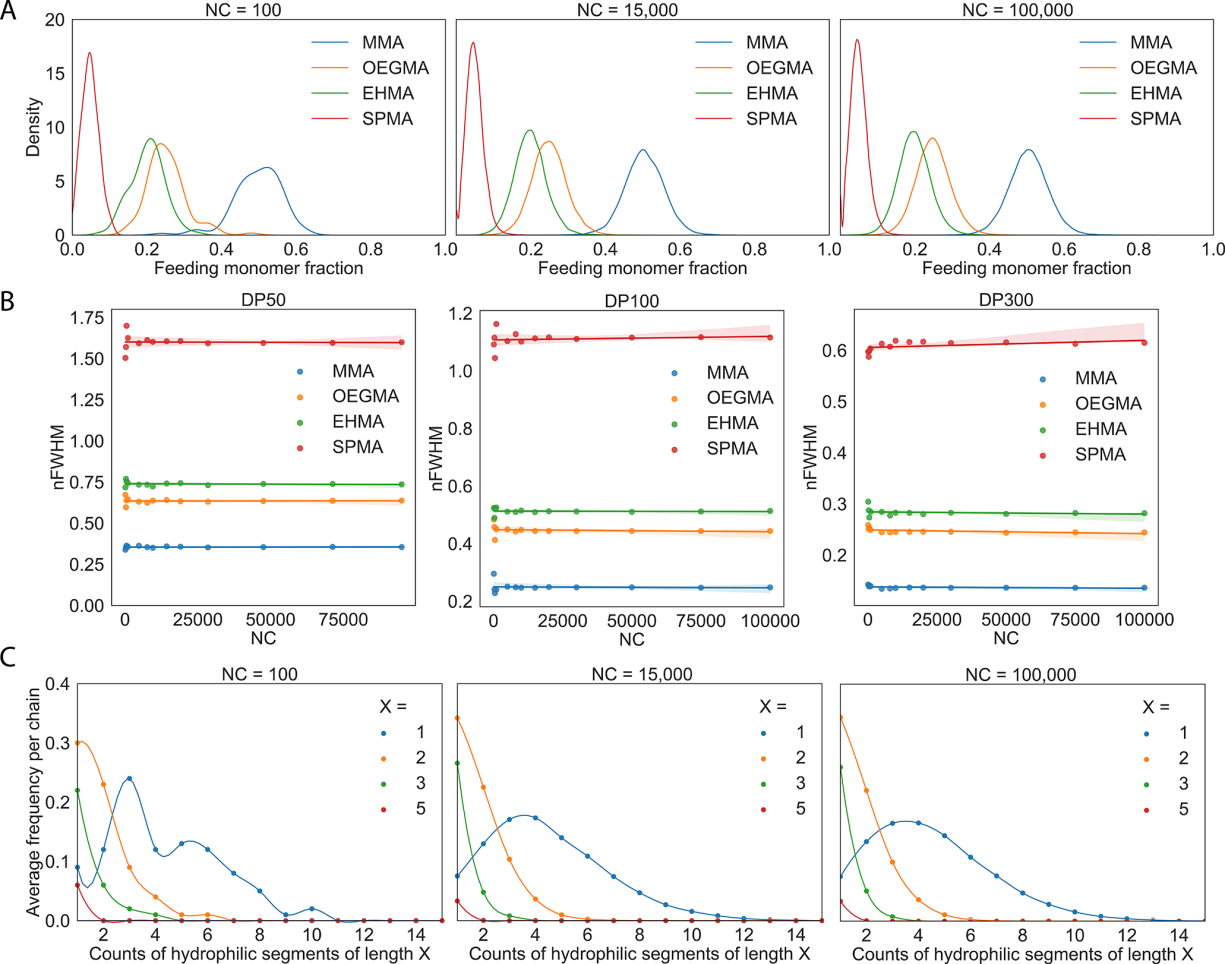
Varying the number of chains (NC) simulated, for a simulated 4 monomer RHP of 50% methyl methacrylate (MMA), 25% poly(ethylene glycol) average *M*_n_ 500 (OEGMA), 20% 2-ethylhexyl methacrylate (EHMA), and 5% 3-sulfopropyl methacrylate potassium salt (SPMA). (A) Sequence level monomer distributions for batches of NC = 100, 15000, and 100000 (left to right). (B) Normalized full width at half-maximum (nFWHM) plots of batch level monomer feed ratio distributions for increasing degree of polymerization (DP) of 50, 100, and 300 (left to right). (C) Sequence level hydrophobic segment distributions, highlighting segments of lengths 1, 2, 3, and 5 for batches of NC = 100, 15000, and 100000 (left to right).

**Figure 3:**
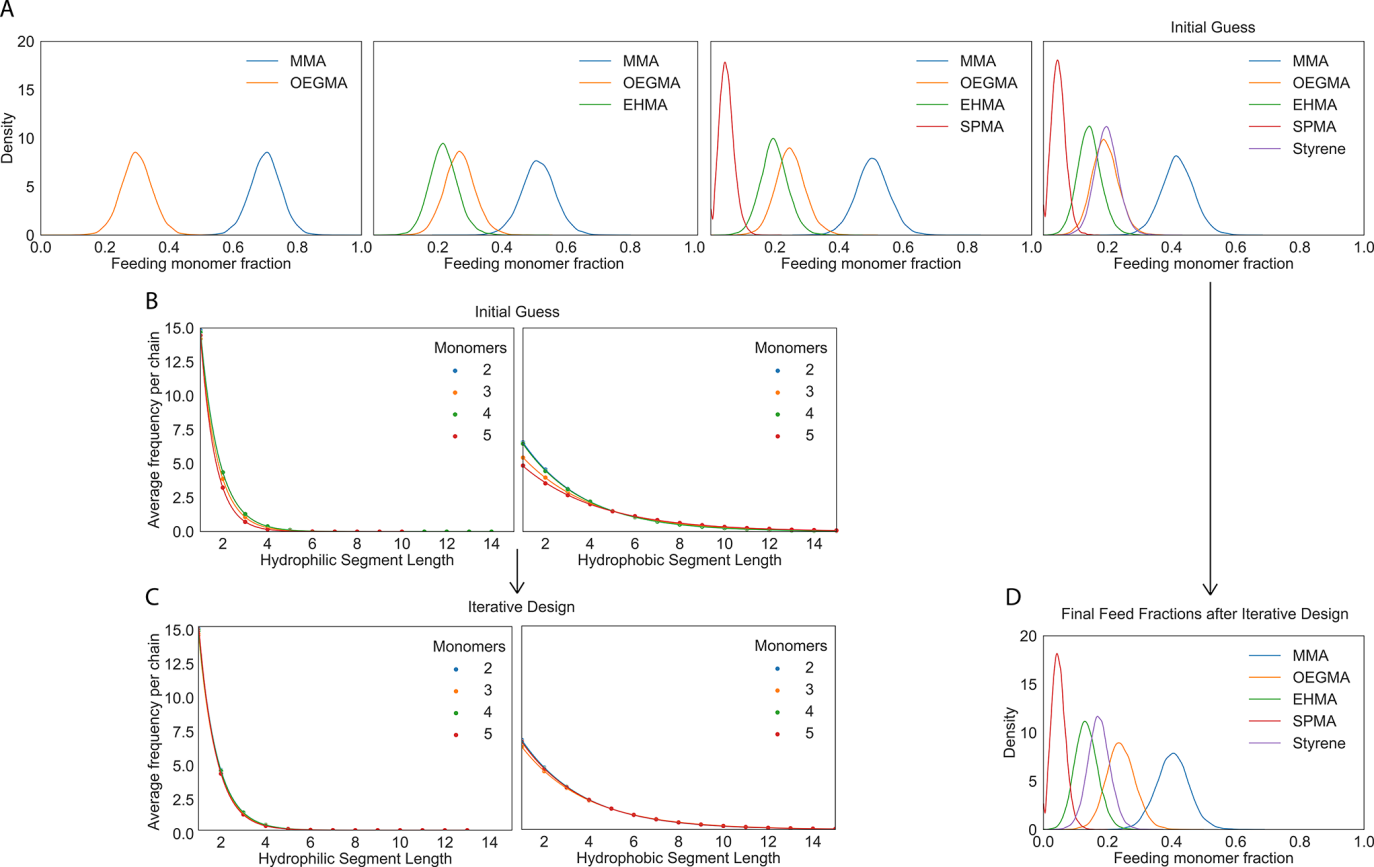
Varying the number of unique monomers. For simulated RHP batches of average degree of polymerization 100 with varying number of unique monomers from 2 to 5 of methyl methacrylate (MMA), poly(ethylene glycol) average *M*_n_ 500 (OEGMA), 2-ethylhexyl methacrylate (EHMA), and 3-sulfopropyl methacrylate potassium salt (SPMA). (A) Sequence level monomer distributions for each simulated batch with initial guess monomer feeding ratios of 70:30, 51:27:22, 50:25:20:5, and 45:20:15:5:15 (left to right). (B) Batch level hydrophilic and hydrophobic segment distribution heterogeneities of initial guess monomer feeding ratios. (C) Batch level hydrophilic and hydrophobic segment distribution heterogeneities after iterative adjustment of monomer feeding ratios to 70:30, 50:29:21, 50:25:20:5, and 43:25:14:5:13 (left to right). (D) Sequence level monomer distributions for iteratively designed 5 monomer RHP with 43:25:14:5:13 feeding ratio.

1. *Chemical heterogeneity*: analysis of how monomers along the sequence vary. Subsets of sequences from simulated batches are visualized for segment level comparison along a single chain (Figure 1a). On the sequence level, kernel density estimate (KDE) plots show the distribution of monomers across all chains in a single batch. A curve fitted to a histogram is plotted for each unique monomer to show the distribution of monomer fractions on each chain in a single batch of simulated polymer (Figure 2a). For each batch, the full width at half-maximum (FWHM) of the peaks in KDE plots is calculated, normalized by each monomer feeding fraction (nFWHM), and visualized on a single scatter-line plot (Figure 2b). The *x*-axis of nFWHM batch-to-batch level comparison plots can be varied to probe trends across different input parameters such as number of sequences and average degree of polymerization.
2. *Segmental hydrophobicity*: sequences are binarized into hydrophobic or hydrophilic monomers and grouped into segments. The hydrophile–lipophile balance (HLB) value was used to evaluate the solubility of monomer side-chains through group contribution theory. Using the equation HLB = 7 + ∑_*i*_*n*_*i*_HLB_*i*_, where *n*_*i*_ is the number of the *i*th chemical group in the molecule with corresponding value HLB_*i*_.^63^ The HLB value for each monomer side chain used in this work was estimated as HLB[methyl methacrylate (MMA)] = 8.45, HLB[2-ethylhexyl methacrylate (EHMA)] = 5.12, HLB[poly(ethylene glycol) average *M*_n_ 500 (OEGMA)] = 11.4, HLB[3-sulfopropyl methacrylate potassium salt (SPMA)] = 18.5, and HLB[styrene (STY)] = 4.865. Lower HLB values indicate higher hydrophobicity, and a higher value means greater hydrophilicity. A hydrophilic–hydrophobic cutoff value (HLB-threshold) of 9 was set to distinguish hydrophobic and hydrophilic monomers. A hyrophobic (or hydrophilic) segment is considered to be a contiguous run of hyrophobic (or hydrophilic) monomers. These segments can be visualized on sampled subsets of binarized sequences (Figure 1b). Sequence level heterogeneity is analyzed by counting the hydrophobic segments of each block length on each chain. The number of hydrophobic segments is then averaged per chain. For visualization, the average frequencies per chain for select segment lengths (i.e., 1, 3, 5, and 10 monomers long), which is the same as the block length for this analysis, are plotted (Figure 2c). Segment length and count frequencies on each chain can also be summed (rather than averaged) across all chains in a batch for a batch level heterogeneity comparison of total segment distributions (Figure 3b).
3. *Sliding window analysis*: sliding window analysis is routinely used for protein sequence analysis to reduce random noise and obtain coarse-grained but more obvious characteristics at the segment level. We thus applied a level of convolution to RHP sequences prior to segmental hydrophobicity analysis. Average segmental HLB values are continuously calculated for a window sliding from the alpha to the omega ends of the simulated RHP chains. The window is advanced by one monomer each time. We used a span containing odd numbers of monomers and assigned the average HLB value of that span to its middle monomer. Various window sizes of 5, 9, and 15 were adopted from previous works to study the effects of small, medium, and large numbers of neighbor monomers, respectively.^57−60^ Hydropathy plots were generated to visualize randomly sampled sequences for each RHP composition and window size (Figure S1). The hydrophobic/hydrophilic segments were identified using the same definition from Metric 2, except that the HLB value for each monomer was replaced with window-averaged values. At the sequence level, hydropathy plots were averaged across all chains within a batch and distribution statistics were plotted (Figure S2).
4. *Specific segment search*: distributions of specific segments of interest are analyzed. This metric is similar to Metric 2 in that sequence level analysis averages chain distributions and batch level comparisons sum the distributions. However, instead of hydrophobic/hydrophilic segments, segments are specifically defined by a desired chemical (monomer) pattern. An example module has been implemented that searches for hydrophobic segments containing 1 embedded OEGMA monomer that is 2 or more monomers away from the end of the segment. For sequence level analysis, manually selected segment lengths of 5, 8, 10, and 13 are plotted. For batch level analysis, kernel density distribution of the specific segments for the given batch are plotted (Figure 5c).

Any combination of evaluation metrics may be used for a given sequence analysis. To generate reaction schematics that physically realize this desired sequence distribution, the corresponding input parameters and additional information about the reaction scale, monomer molecular weight, monomer density, initiator, chain-transfer agent, and solvent, are solved in a system of equations. Accordingly, the required volumes and masses of each reagent are output.

### Protein Sequences

Protein sequences can be analyzed through the same workflow for direct comparison to the RHP sequences. A dictionary is created mapping amino acids into groups of roughly corresponding RHP monomers by hydrophobicity/hydrophilicity/charge. The dictionary is applied to convert protein primary sequences into RHP-monomer equivalent sequences—a dimensionality reduction from an alphabet size of 20 (AA residues) to between 2 and 5 (synthetic monomers). A specific protein sequence is then split into overlapping segments governed by a segment length = 100 and offset (spacing between segment start monomers) = 10. An example is shown in Figure 1c. These segments are passed in as simulated RHP-sequence equivalents into the workflow for direct comparison.

### Software

All code used for calculation and visualization in this work are provided as an open-source repository (https://github.com/ivanjayapurna/RHPapp), and key features have been implemented as a web application (https://www.ocf.berkeley.edu/xugroup/rhpapp) to serve as a tool for community use.

## Results and Discussion

### Simulation Scale and Fidelity

The entire premise of RHP design by simulation is based on the assumption of a controlled link between synthetic design and actualized statistical monomer distribution. RHP sequence simulation can only be insightful when a polymerization retains its livingness such that synthesis is predictable. To experimentally verify synthetic control, we conduct routine characterizations on synthesized materials by nuclear magnetic resonance spectroscopy (NMR) and gel permeation chromatography (GPC). With these two common characterization techniques we confirm in our samples (1) bounded polydispersity that confirms good RDRP control, (2) reaction conversion percentage that confirms no compositional drift, (3) achieved targeted molecular weight, and (4) approximate composition percentages (Figure S3). However, an important caveat is that both in-lab and in-silico experiments only probe a tiny subpopulation of statistically possible RHP sequences. To illustrate the scale of our materials in number of polymer chains, let us assume the synthesis of 1 g of a hypothetical methacrylate-based RHP, with an average monomer molecular weight of 100 g per mole and an average degree of polymerization (DP) of 100. This would yield on the order of 10^18^ polymer chains synthesized. GPC or NMR will use on the order of 1 mg of sample, which is on the order of 10^15^ chains. Thus, when characterizing with GPC or NMR, we make the assumption that an approximately 0.1% sample is representative of the total batch synthesized. A similar approximation will need to be made computationally.

A key parameter in stochastic (Monte Carlo) simulators is the minimum sample size required for simulated results to converge to a stable value. For the RHPapp, the key parameter is the number of chains simulated (NC). Computational performance limits our ability to simulate 10^18^ chains, an approximate magnitude of a real synthesis of a batch of RHP. A similar subsampling approximation as was made for experimental characterization (roughly 3 orders of magnitude lower) must be done for in-silico characterization. In a good stochastic simulation of polymerization, results should converge to the same, stable value regardless of how many sequences are simulated. The optimal NC is the minimum required to converge to a stable distribution of results to maximize simulation accuracy and minimize computational time. As an example to illustrate finding an NC minimum for a batch of 4-monomer methacrylate-based RHPs of DP of 100, we simulated multiple batches with varied NC while keeping all other simulation parameters fixed.

Using chemical heterogeneity as a metric, there is visible lack of smoothness in the KDE fitting when only 100 sequences are simulated, when compared to 15000 and 100000. However, the important high-level features across all 3 batches of varying NC such as peak location, height, and width remain similar (Figure 2a). The normalized full width at half-maxima (nFWHM) of all peaks from the KDE plot were estimated (Figure 2b). For an average DP of 100, although the differences in chemical heterogeneity are minor between the number of sequences simulated, the monomer of lowest feed ratio (SPMA) has a convergence of initial nFWHM oscillation at around NC = 15000. This minimum threshold is approximately 14 orders of magnitude lower than a real experimental RHP synthesis and is acceptable as it requires minimal compute power to simulate at NC ≥ 15000. The minimum NC is a parameter intrinsically linked to co-input parameters and may vary significantly when other parameters change, such as molar feed ratios or number of monomers. However, in some cases, such as increasing or decreasing average DP of our example system and keeping all else constant, the minimum NC is similar. Although the actual nFWHM values for average DP 50 and 300 differ significantly from those of DP 100, all initial oscillations stabilize at a similar NC threshold.

The minimum NC can also be estimated using sequence hydrophobic/philic segmental distributions, as seen in the binarized sequences in Figure 1b. Comparing different NC simulations at the sequence level within batches of DP 100 RHPs, there is no shift in the primary peak mean or heights in the average frequency per chain distributions of hydrophobic and hydrophilic segments. However, there is a noticeable gain in smoothness of fits and disappearance of misleading minor peaks as NC increases (Figure 2c). As the increase in fit smoothness is negligible between 15000 and 100000 NC, the segmental hydrophobicity metric at the sequence level suggests NC = 15000 is sufficient for stable, accurate simulation distributions. Analysis at the batch level shows a negligible difference in batch level frequency and distribution of segments normalized by number of sequences (Figure S4). Thus, NC was set to 15000 for all 3 subsequent example use-cases using the same 4 monomer methacrylate RHP presented in this work.

### Sequence Analysis to Guide Random Heteropolymer Design

Panganiban et al. proposed that 4 monomer RHPs can stabilize proteins in aqueous and organic solutions when they have both (1) chemical heterogeneity and (2) hydrophobic/hydrophilic block length and count distributions that mimic those of intrinsically disordered proteins.^25^ We used simulated RHP sequences to decouple these two hypotheses and assist in the design of RHP sequences of a varying number of unique monomers that still retain the same hydrophobic/hydrophilic segment distributions as the original 4 monomer RHPs. The two fixed monomers are MMA and OEGMA, with EHMA added for monomer 3, SPMA for monomer 4, and STY for monomer 5. Styrene was chosen to represent a common non-methacrylate monomer to demonstrate the potential for monomeric diversity in this design framework. Initial guesses of appropriate monomer feeding ratios for each RHP were made to approximate similar hydrophobic/hydrophilic segment length and count distributions. Figure 3a shows that the sequence level chemical heterogeneities of RHP batches of varying monomers are vastly different. However, despite the disparity in chain chemistry, at the binarized hydrophobic/hydrophilic level, the segmental distributions for the 2 and 4 monomer RHP batches are nearly identical, suggesting no need to alter initial guess monomer feeding ratios (Figure 3b). This evaluation metric was used to further fine-tune the designs of the 3 and 5 monomer RHPs for a more precise distribution match. An iterative design approach on our initial guesses of the 3 and 5 monomer RHPs enabled precise monomer feeding ratio alterations to preserve the targeted chemical heterogeneity (Figure 3d) while minimizing the disparity in theoretically predicted hydrophobic/hydrophilic segment length distributions between each RHP batch (Figure 3c). Thus, when designing an RHP synthesis for a materials application, analysis of simulated RHP sequences can serve as an in-silico prescreening, to inform and accelerate the rational design of compositions to experimentally characterize.

### Importance of Robust Segment Definition

**Figure 4:**
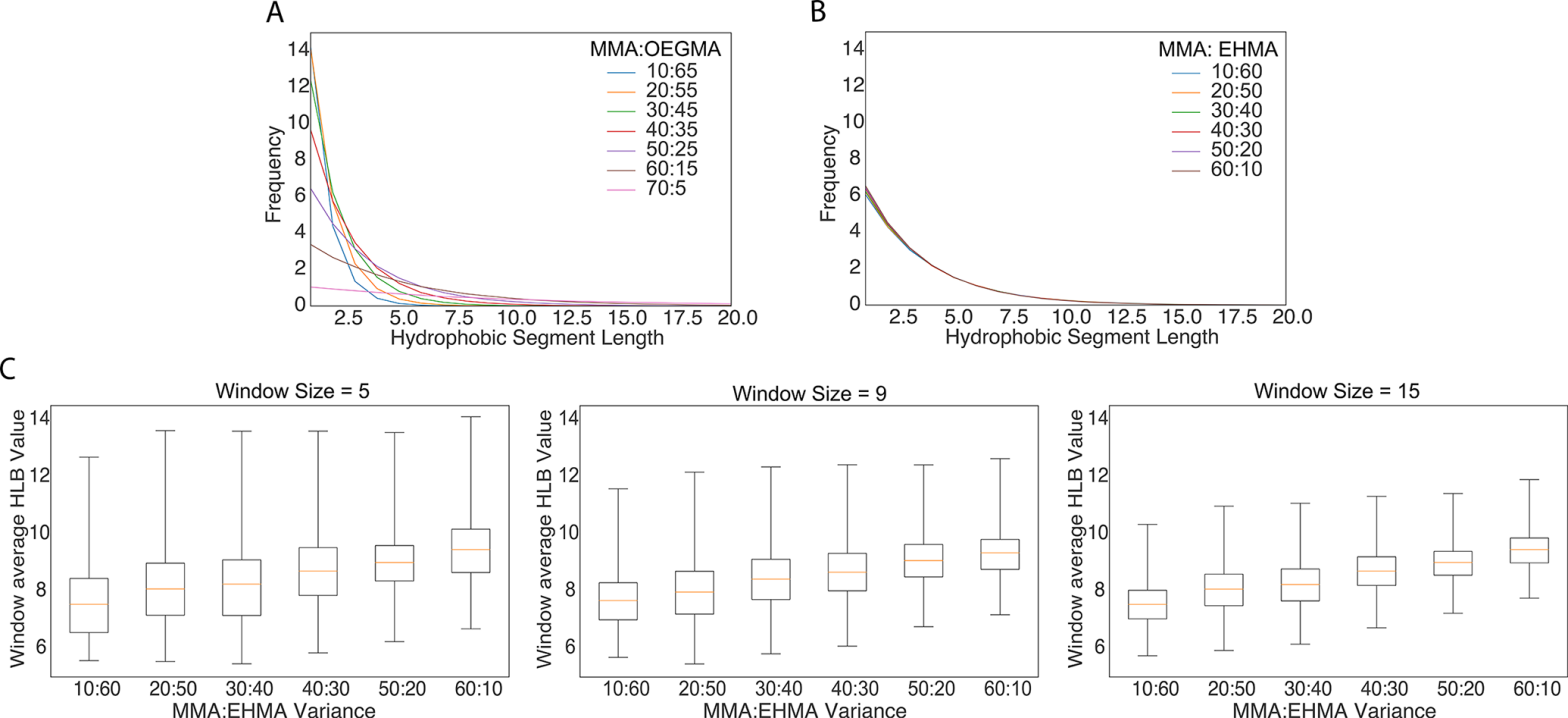
Varying the monomer feed ratio. Batch level hydrophilic segment distribution heterogeneities for a simulated 4 monomer RHP of methyl methacrylate (MMA), poly(ethylene glycol) average *M*_n_ 500 (OEGMA), 2-ethylhexyl methacrylate (EHMA), and 3-sulfopropyl methacrylate potassium salt (SPMA) of degree of polymerization 100: (A) varying MMA:OEGMA feed ratios and fixed 20% EHMA and 5% SPMA; (B) varying MMA:EHMA feed ratios and fixed 25% OEGMA and 5% SPMA. (C) Batch level statistics after sliding windows of sizes 5, 9, and 15 (left to right) are applied, and the resulting sequence level segment information is averaged by sequence position.

DelRe et al. demonstrated that RHPs can nanoencapsulate and preserve the activity of enzymes in solid polymeric matrices. RHP composition was shown to regulate substrate binding and active site availability.^4^ However, only a few RHP compositions were tested, due to the lack of high throughput material synthesis and characterization. Simulated sequence analysis can assist in data deficient modeling, analysis and serve as a useful tool to suggest explanations for experimental findings. The 4 monomer RHP used has 2 hydrophobic and 2 hydrophilic monomers, giving several handles to tune. The first we chose to modulate is the MMA:OEGMA ratio. MMA is our proxy for segmental hydrophobicity and OEGMA for segmental hydrophilicity. Tuning this handle yields clear differences in segment length distributions (Figure 4a) that could be compared to differences in enzyme nanoencapsulation behavior based on enzyme surface hydrophilic and hydrophobic patch distribution patterns.

However, polymer–protein interactions are sensitive and complex. Rather than leading to improved performance, too drastic of a change in RHP composition could overshoot the scale of differentiation between enzyme chemical distributions resulting in worse chaperone performance or even polymer gelation issues as not all RHP compositions can be synthesized.^29^ To tune with higher sensitivity the MMA:EHMA ratio can be varied. Although both of these monomers are considered hydrophobic by the HLB threshold parameter of 9, in reality this binarization is just an artifact of analysis. A lower HLB threshold that would split EHMA and MMA could be chosen that would yield different results. In the current analytical setup, adjusting the MMA:EHMA ratio has no apparent effect (Figure 4b), contrary to experimental results. To more subtly fine-tune using a method that is more robust to threshold parameter selection, we redefine what it means to be a hydrophobic/hydrophilic segment. A level of sliding window convolution prior to binarizing into contiguous hydrophobic/hydrophilic segments adds an abstraction layer from monomer specificity, which is inherently stochastic and noisy. Sliding window analysis allows us to loosen the rigid prior definition of what is considered a segment. The results of the analysis suggested that, within a batch, window average HLB distributions are invariant to central monomer position along the chain, with the exception of increased variance at the omega end of simulated chains, where due to polydispersity there are fewer data points to average and converge to the expected statistical distribution (Figure S2). Thus, sequences can be further averaged across positions along the chain to make cleaner batch-average segmental (window) hydrophobicity comparisons. Differences can be observed in batch level segmental distribution statistics, where segments are now defined by window average HLB values (Figure 4c). The mean values and trend of increasing window average HLB as the MMA:EHMA ratio increases are consistent across varying window sizes. Although variance reduces with increasing window size, here we have demonstrated that applications where we primarily consider the resulting trends in average values, such as this RHP–enzyme interaction analysis, are invariant and thus robust to the range of window sizes selected.

### Protein and RHP Sequence Comparison

**Figure 5:**
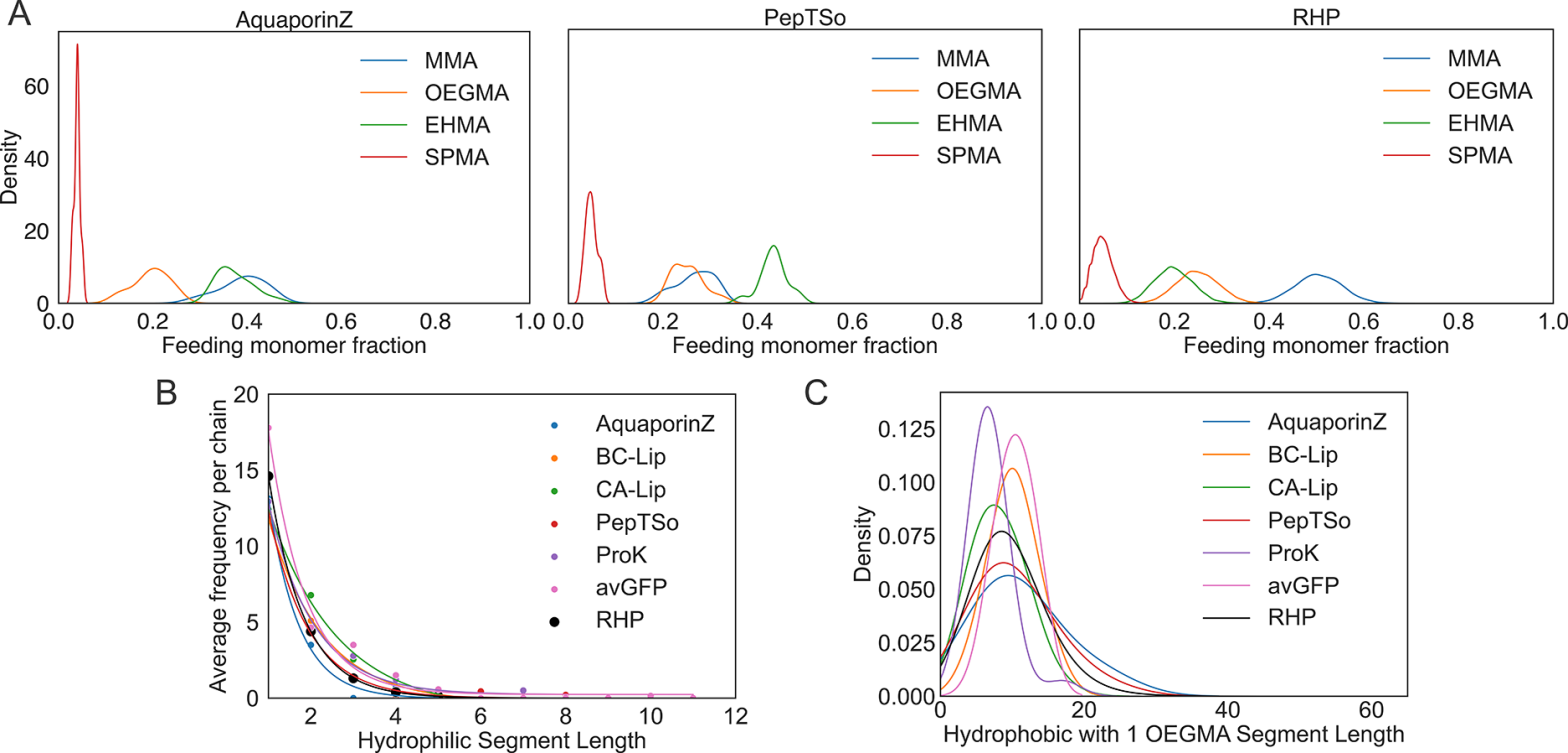
Protein and RHP comparison. 6 proteins were convolved into RHP sequence space, segmented to form a batch, and analyzed through the RHPapp. Proteinase K (ProK), lipase from *Burkholderia cepacia* (BC-Lip), lipase from *Candida antarctica* (CA-Lip), Aquaporin Z (AquaporinZ), POT family transporter (PepTSo), and green fluorescent protein from Aequorea victoria (avGFP) were compared to a simulated 4 monomer RHP of 50% methyl methacrylate (MMA), 25% poly(ethylene glycol) average *M*_n_ 500 (OEGMA), 20% 2-ethylhexyl methacrylate (EHMA), and 5% 3-sulfopropyl methacrylate potassium salt (SPMA). All sequences are of average degree of polymerization 100. (A) Sequence level monomer distributions for AquaporinZ, PepTSo, and RHP (left to right). (B) Batch level hydrophilic segment distributions. (C) Batch level kernel density estimate of hydrophobic segments with 1 OEGMA per chain.

Protein sequences can be convolved into corresponding RHP sequence space, segmented to form a batch, and then analyzed through the RHPapp workflow for direct comparison to RHP sequences. Six sample proteins (3 enzyme hydrolases, 2 transmembrane proteins, and 1 β barrel structure protein) were analyzed through the RHPapp to demonstrate facile comparison to a 4 monomer methacrylate-based RHP (Figure S6). On the sequence level, proteins each have characteristic chemical heterogeneities despite functional and evolutionary similarity. Membrane proteins AquaporinZ and PepTSo have different chemical heterogeneities despite similar function (Figure 5a). Despite the chemical heterogeneity differences, all 6 proteins display similar hydrophobic segment distributions on the sequence level (Figure 5b). This may suggest a degree of generality in the design of RHPs, as was demonstrated by Panganiban et al. where a single RHP design stabilized various proteins in solutions. Chemical heterogeneity differences may explain why DelRe et al. observed that different RHPs were required for optimal nanoencapsulation of different hydrolases (Figure S5).

In addition to interfacing with proteins as binders, Jiang et al. showed RHPs can independently mimic membrane protein function to undergo rapid and selective proton transport across lipid bilayers at a rate similar to those of natural proton channels.^26^ Specific RHP segments of critical importance to recapitulating transport function are hydrophobic segments containing 1 embedded OEGMA monomer that is 2 or more monomers away from the end of the segment. All functional chains contained this pattern within their random sequence. This functional RHP of DP 100 has a different chemical heterogeneity profile compared to both membrane proteins Aquaporin Z and PepTSo (Figure 5a), suggesting global chemistry is nonessential to mimicking protein function. A more local analysis of the proton transport specific segment pattern was done comparing the RHP to 6 proteins at the batch level (Figure 5c). Of the sampled proteins, only the 2 membrane proteins display a similar distribution to the functional RHP. Specifically, the distributions suggestive of selective proton transport function display a lower density peak at specific segment length 9 and a longer tail with sizable population between 20 and 30 segment length. This example demonstrates the comparison of statistical distributions of specific segment motifs on RHPs to those on proteins to explain experimentally the observed protein-like function.

## Conclusion

We demonstrate a viable path toward guiding the rational design of RHPs as synthetic protein mimics through the combination of RDRP polymerization simulation and bioinformatics-inspired sequence analysis. The RHPapp, more than an open-source toolkit, is a design and analysis approach that can be applied to a diverse range of impactful projects well beyond the methacrylate backbone monomers presented herein. More complex random heteropolymers with monomers of varied reactivity ratios could further enhance control by incorporating partial blocky features into the statistical randomness to create more complex repeating motifs similar to those found in natural proteins. Mirroring the nature of RHPs, the RHP design approach and software presented are general in principle for broad applicability, but also modular and easily fine-tuned to suit projects with exact specificity. Our vision is for the RHPapp to take as input a protein sequence and desired function as a starting point, from which an ideal sequence distribution to target can be designed and translated into controllable RDRP synthetic parameters to be experimentally realized. This analysis framework for simulated heteropolymer sequences couples powerfully with advances in high throughput synthesis and characterization. Just as bioinformatics, the inspiration for our work, has trended toward the realms of big data and more sophisticated statistical modeling techniques, analysis, and machine learning, we propose that this emergent field of macromolecular cheminformatics is ripe to follow suit.

## Supporting Information Available

Examples of segment and sequence level sliding window analysis of RHP sequences, NMR and GPC RHP characterization; supplementary figures for analysis of varying number of chains and comparison of proteins and RHPs; table of reactivity ratios (PDF)
