# Supplementary material for: Sequence Design of Random Heteropolymers as Protein Mimics
Source: Biomacromolecules. 2023 Jan 13;24(2):652–60. doi: 10.1021/acs.biomac.2c01036 (PMC9930114; doi:10.1021/acs.biomac.2c01036)
Supplement: Supplementary file 1 — bm2c01036_si_001.pdf [file bm2c01036_si_001.pdf]

Sequence Design of Random Heteropolymers as Protein Mimics – Supporting Information

Ivan Jayapurna[1], Zhiyuan Ruan[1], Marco Eres[2], Prajna Jalagam[1], Spencer Jenkins[2], and Ting Xu[*,1,2,3]

1. Department of Materials Science and Engineering, University of California, Berkeley, CA, 94720, USA
2. Department of Chemistry, University of California, Berkeley, CA 94720, USA
3. Materials Sciences Division, Lawrence Berkeley National Laboratory, Berkeley, CA, 94720, USA

***Corresponding Author: tingxu@berkeley.edu**

Table S1 | Table of reactivity ratios (Row-Col reactivity) used in this work. Taken from the RHPapp online database. Tabulated reactivity values should be read as $RR_{RowCol} = k_{RowRow} / k_{RowCol}$. All self-additions are assumed to be 1, therefore when $RR_{RowCol} < 1$, for growing polymer chain where the row monomer is terminal, the column monomer is more likely to add on than another row monomer.

	MMA	OEGMA500	2EHMA	3SPMA	STY
MMA	1	0.89	0.89	0.89	0.46
OEGMA500	1.09	1	1	1	0.46
2EHMA	1.09	1	1	1	0.46
3SPMA	1.09	1	1	1	0.46
STY	0.52	0.52	0.52	0.52	1

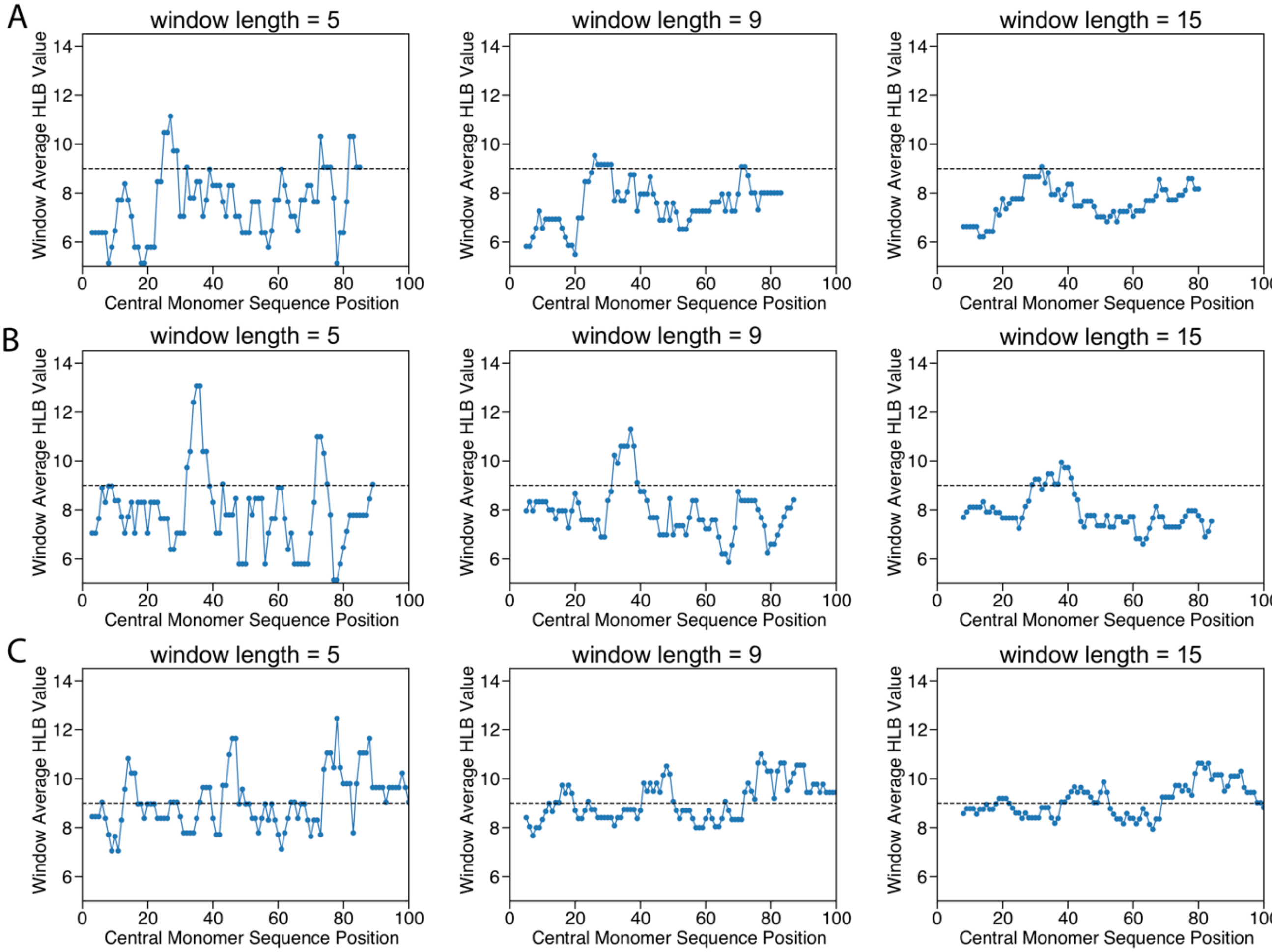

Fig. S1 | Example segment level sliding window analysis of RHP sequences. Sliding window of 3 varying window lengths applied to random sequences sampled from a simulated 4 monomer RHP of methyl methacrylate (MMA), polyethylene glycol average Mn 500 (OEGMA500), 2-ethylhexyl methacrylate (EHMA) and 3-sulfopropyl methacrylate potassium salt (SPMA) of compositions: **A.** 10:25:60:5 **B.** 30:25:40:5 **C.** 60:25:10:5

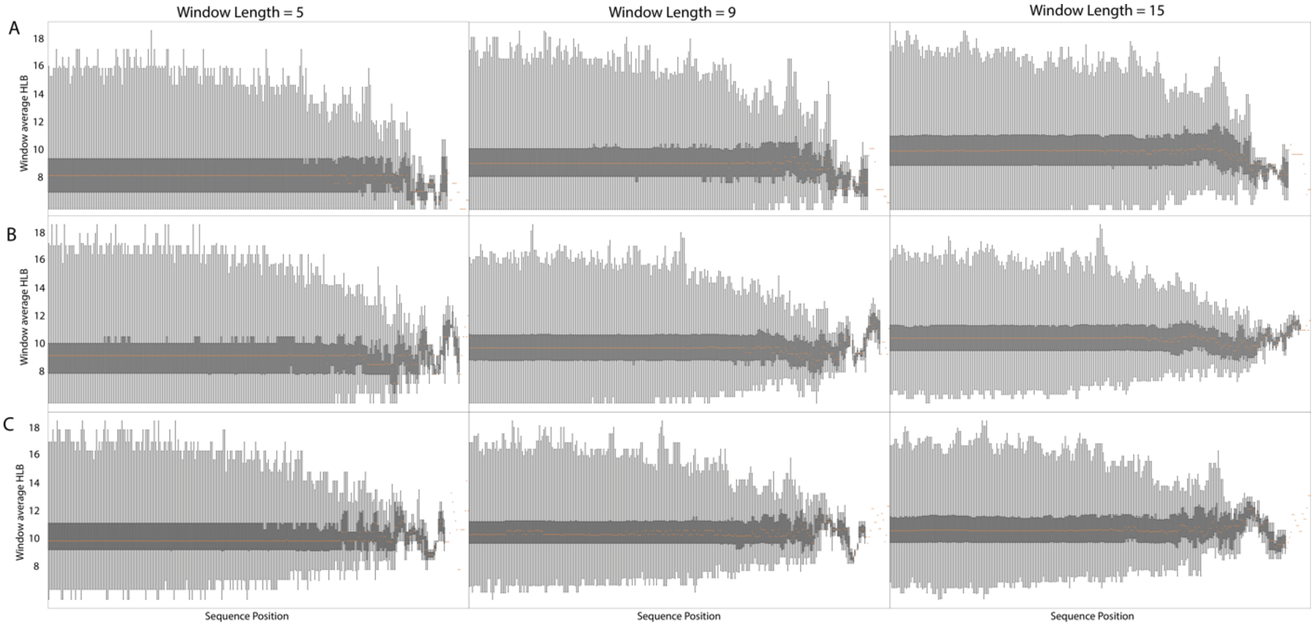

Fig. S2 | Example sequence-level sliding window analysis of RHP sequences. Sliding window of 3 varying window lengths applied to batches of simulated 4 monomer RHP of methyl methacrylate (MMA), polyethylene glycol average Mn 500 (OEGMA500), 2-ethylhexyl methacrylate (EHMA) and 3-sulfopropyl methacrylate potassium salt (SPMA) of compositions: **A.** 10:25:60:5 **B.** 30:25:40:5 **C.** 60:25:10:5

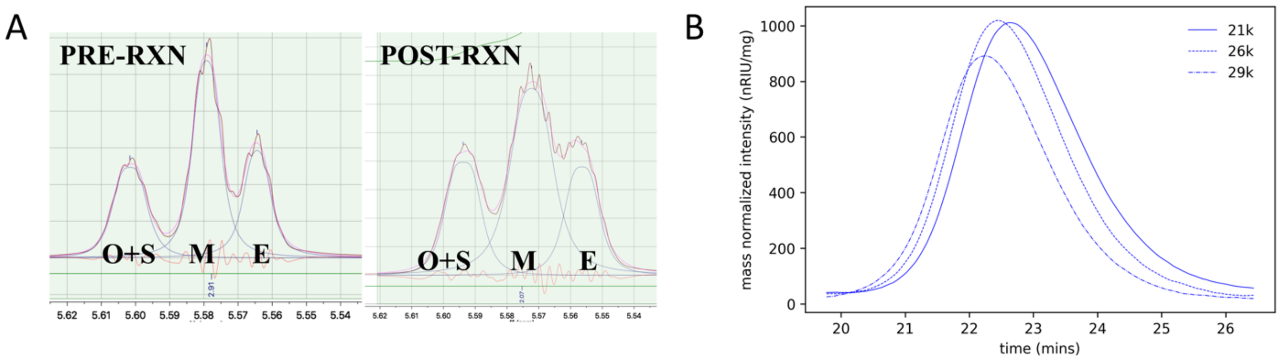

Fig. S3 | Example of routine experimental characterization of RHPs designed by RHPapp. For a 4 monomer RHP of 50% methyl methacrylate (M), 25% polyethylene glycol average Mn 500 (O), 20% 2-ethylhexyl methacrylate (E) and 5% 3-sulfopropyl methacrylate potassium salt (S): **A.** Proton nuclear magnetic resonance spectroscopy (H-NMR) of the methacrylate monomer vinyl peaks pre- and post- reaction samples that can be integrated with respect to an internal standard to verify global conversion and in some cases monomer specific conversions to confirm an absence of compositional drift. **B.** Gel permeation chromatography (GPC) spectra of 3 RHPs of number average molecular weights (Mw) of 21, 26 and 29 kDa that confirm bounded polydispersity and achieved Mw targeted by RHPapp with respect to a polymethyl methacrylate or polyethylene glycol standard.

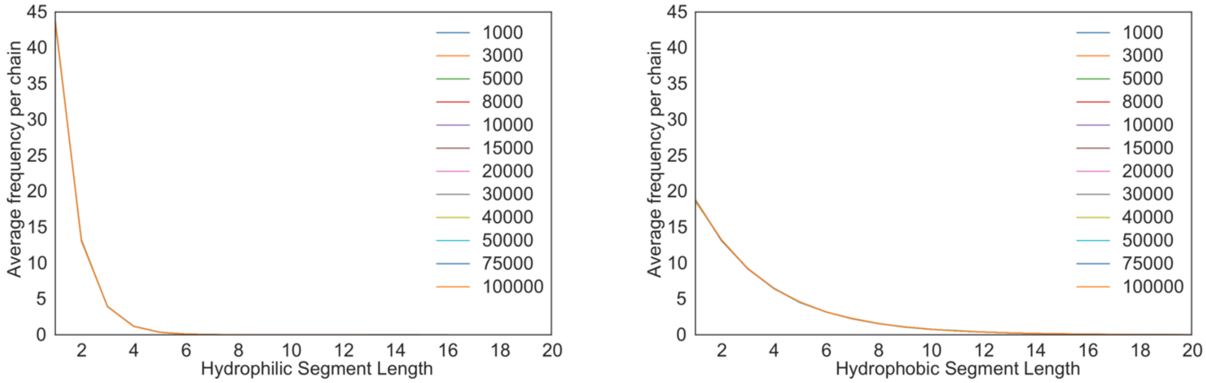

Fig. S4 | Varying the number of chains (NC) simulated. Batch-level hydrophilic and hydrophobic segment distribution heterogeneities for a simulated 4 monomer RHPs of 50% methyl methacrylate (MMA), 25% polyethylene glycol average Mn 500 (OEGMA), 20% 2-ethylhexyl methacrylate (EHMA) and 5% 3-sulfopropyl methacrylate potassium salt (SPMA), of varying NC.

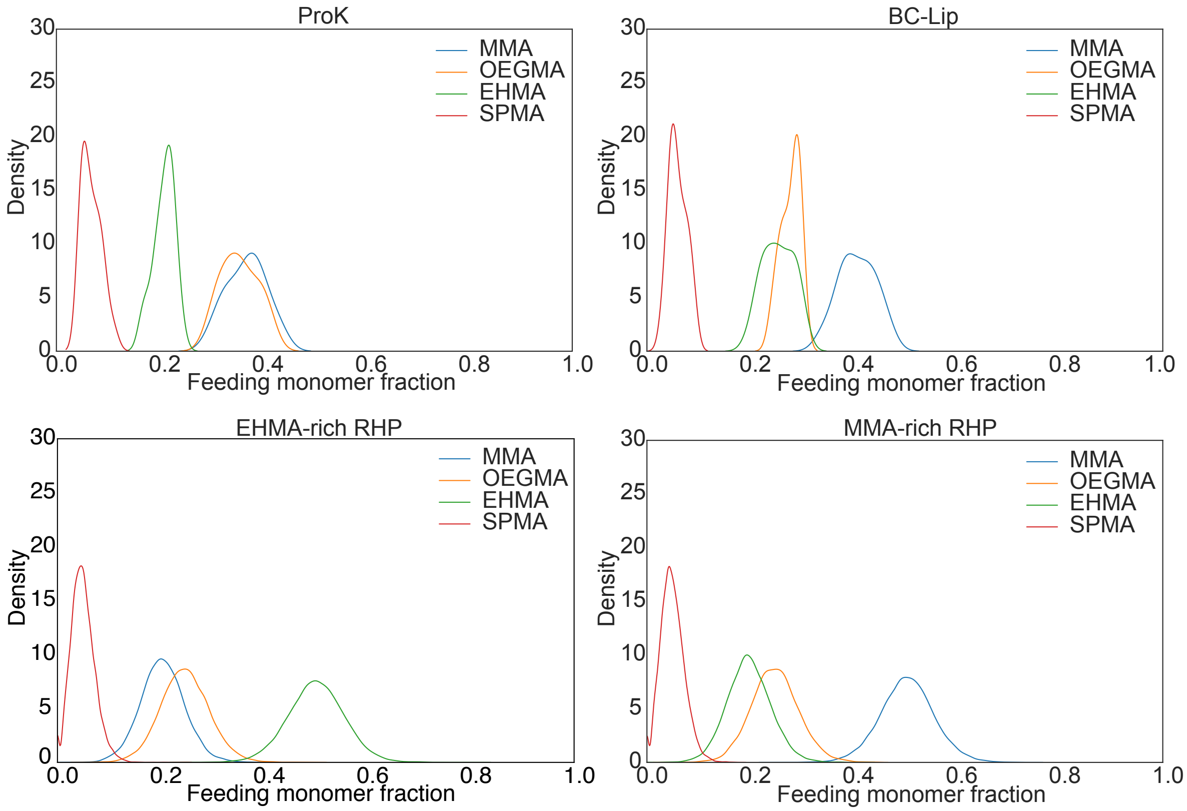

Fig. S5 | Sequence level monomer distributions of Proteinase K (ProK) and Lipase from Burkholderia cepacia (BC-Lip) convolved into RHP sequence space, segmented to form a batch, and 2 batches of simulated 4 monomer RHPs of methyl methacrylate (MMA), polyethylene glycol average Mn 500 (OEGMA), 2-ethylhexyl methacrylate (EHMA) and 3-sulfopropyl methacrylate potassium salt (SPMA) of monomer feeding ratios 50:25:20:5 (MMA-rich) and 20:25:50:5 (EHMA-rich). All sequences are of average degree of polymerization = 100.

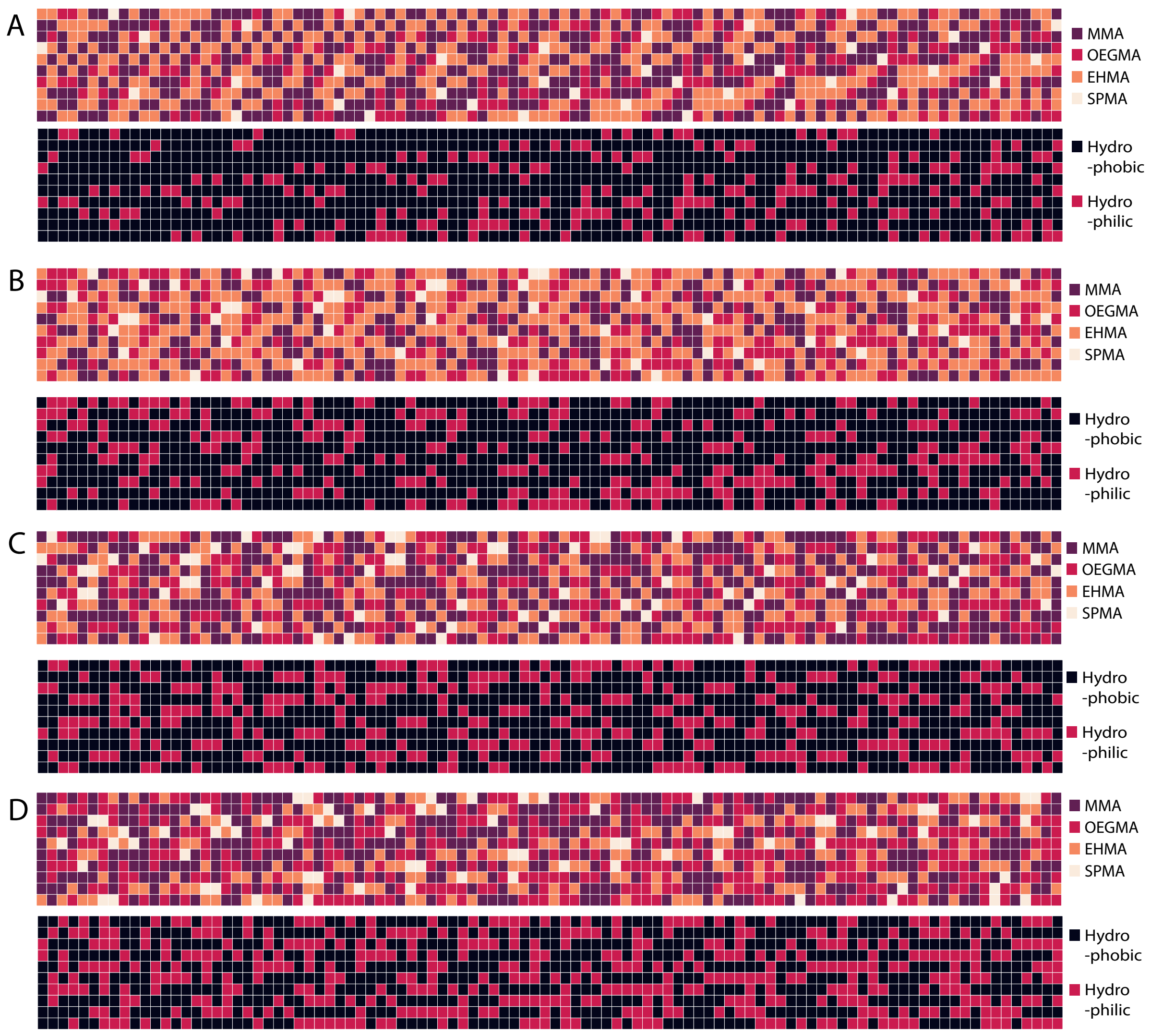

Fig. S6 | Protein sequences in RHP sequence space. The following membrane proteins are segmented and translated to RHP sequence space, then binarized to hydrophobic and hydrophilic units: (A) AquaporinZ (B) PepTSo (C) BC-Lipase (D) Proteinase K
